# When the brain talks back to the eye

**DOI:** 10.1371/journal.pbio.3003454

**Published:** 2025-11-05

**Authors:** Dominic Gonschorek, Thomas Euler

**Affiliations:** 1 Institute of Ophthalmic Research, University of Tübingen, Tübingen, Germany; 2 Werner Reichardt Centre for Integrative Neuroscience, University of Tübingen, Tübingen, Germany

## Abstract

The state of our brain shapes what we see, but how early in the visual system does this start? This Primer explores a new PLOS Biology study which shows that brain state-dependent release of histamine modulates the very first stage of vision – the retina.

## Teaser

The state of our brain shapes what we see, but how early in the visual system does this start? A new study in *PLOS Biology* shows that brain state-dependent release of histamine modulates the very first stage of vision in the retina.

Our perception of the world depends not only on external inputs but also on the brain’s internal state. Whether we are attentive, alert, drowsy, or aroused, the very same stimulus can evoke strikingly different responses depending on the current internal state [1]. Visual processing begins in the retina: not only is light captured and converted into electrical signals by the photoreceptors, but these signals are significantly transformed by postsynaptic circuits before being transmitted by the retinal ganglion cells (RGCs) to downstream visual brain regions. Traditionally, the retina has been regarded as a feed-forward image processor that independently relays visual information to the brain, leaving it to downstream visual stages, such as the thalamus or the superior colliculus (SC), to initially integrate retinal signals with other sources of sensory information and behavioral states [2]. Yet, growing evidence challenges this picture, suggesting that retinal circuits are already dynamically modulated by the animal’s state.

For example, it has been shown that pupil dilation during active behavior shifts photoreceptor recruitment in the retina, rapidly altering color sensitivity in cortical neurons and enhancing the detection of ethologically relevant stimuli [3]. It has been suggested that these arousal-mediated modulations may be directly driven by the retina or by higher cortical activity. Subsequent work demonstrated that through the “pupillary contrast response”, retinal circuits indeed contribute to modulating pupil size, for instance, as a function of visual contrast, supporting interactions between retinal circuits and brain states [4]. Other studies further revealed that the retinal output—measured in the SC and dorsal lateral geniculate nucleus (dLGN), respectively—is actively modulated by the brain’s state, adding further evidence that vision is shaped not only downstream but also at the retina itself [5,6]. These findings highlight that the view of the retina as an independent feed-forward circuit is too simplified. This should not come as a surprise, as it has long been known that centrifugal projections from downstream visual processing stages provide feedback to the retina in all studied vertebrate species [7,8]. These neuromodulatory projections vary from massive (e.g., in birds) to sparse (in mammals, including mice). Despite their sparseness in mice, it was recently demonstrated that the central histaminergic system, located in the mouse hypothalamus, projects to the retina, where it modulates the activity and feature selectivity of RGCs in *ex vivo* retina and in anesthetized animals [8].

**Fig 1:**
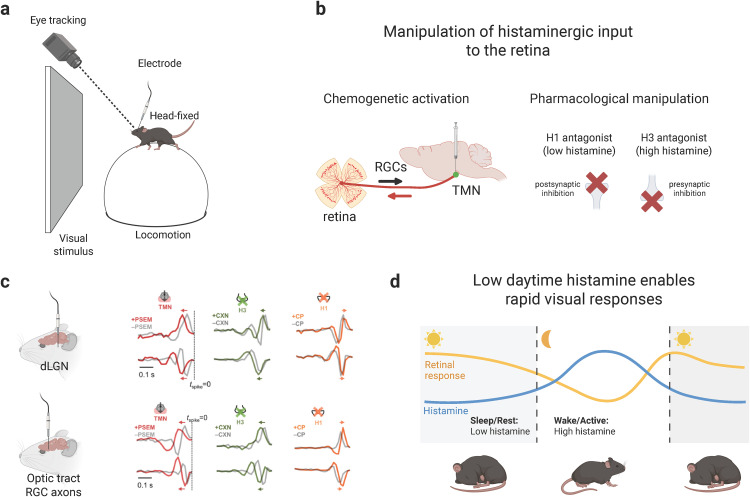
Histamine modulates early visual processing in awake mice. **(a)** Visual responses were recorded in awake, head-fixed mice using single-unit extracellular recordings from retinal ganglion cell (RGC) axons in the optic tract and from dorsal lateral geniculate nucleus (dLGN) neurons, while pupil dynamics and locomotion were monitored. **(b)** Tripodi and Asari manipulated histaminergic input to the retina by chemogenetic activation of hypothalamic tuberomammillary nucleus (TMN) neurons or by systemic pharmacology targeting histamine receptors. H3 receptor antagonists increase histamine release, whereas H1 receptor antagonists block postsynaptic histamine effects. **(c)** Increasing histamine levels, either chemogenetically or pharmacologically, slowed and weakened visual responses in both RGC axons and dLGN neurons, whereas blocking H1 receptors had the opposite effect, consistent with H1 receptor-mediated gain modulation. **(d)** Ethological implication: in nocturnal mice, histamine levels are low during daytime, when the animals rest, enabling faster and stronger retinal responses and potentially facilitating rapid detection of threats. At nighttime, when the animals are active, high histamine levels suppress and slow visual responses. Partly created with biorender.com.

In this issue of *PLOS Biology*, Tripodi and Asari [9] take the crucial next step by examining histamine’s role under awake, more physiological conditions by recording from RGC axons in the optic tract and relay neurons in the dLGN of head-fixed mice (Fig 1a). To control histamine effects, they combined chemogenetic activation of hypothalamic histaminergic neurons with pharmacological manipulation of two main histamine receptors, H1 and H3 (Fig 1b). Simultaneously, they monitored behaviorally relevant cues such as pupil dynamics, pupil size, and locomotion to test whether any observed histamine-induced effects were secondary to arousal-linked behaviors. The authors found that with increasing histamine levels, visual responses in both RGCs and dLGN neurons were consistently slowed and weakened, while blocking H1 receptors had the opposite effect (Fig 1c). Computational modeling suggested that this reflected gain modulation within retinal circuits, thus already at the first step of vision. Importantly, these effects were independent of pupil changes or locomotion, indicating that histamine acts as a direct neuromodulator of early visual circuits rather than indirectly through arousal-linked behaviors.

These findings seem to contradict earlier work, as Warwick and colleagues [8] reported that histamine rather enhanced retinal responses: they showed, for instance, that the responses of direction-selective RGC types became faster and more sharply tuned to motion direction. In contrast, Tripodi and Asari revealed that histamine mediates a broad suppression of responses across cell types in awake animals. This highlights how experimental context—*ex vivo* versus *in vivo*, anesthetized versus awake—may affect the observed results. For instance, *in vivo* recordings from the optic tract revealed that retinal output dynamics differ significantly between anesthetized and awake mice, with awake responses being faster, less variable, and accompanied by markedly higher firing rates [10]. In line with these results, Liang and colleagues [6] showed that arousal-driven modulation of retinal axons in the dLGN is always measurable in awake animals, even in low-arousal states, yet completely absent under anesthesia. Together, these studies emphasize that findings in anesthetized animals or isolated retinae need to be carefully treated—at least in the scope of neuromodulation. Moreover, these findings underscore that neuromodulatory effects in the early visual system, including the retina, are profoundly state- and context-dependent.

By showing that histamine dampens, rather than enhances, early visual responses in awake mice, Tripodi and Asari uncover a surprising role for this neuromodulator. Since histamine levels peak during active wake states, their findings suggest that lower histamine may actually facilitate faster retinal responses. Such facilitation could be ethologically advantageous across species, not just in nocturnal animals, by allowing rapid detection of visual threats during periods of quiescence or reduced activity (Fig 1d). Importantly, Tripodi and Asari also observed that locomotion and pupil dilation can accelerate retinal responses even when histamine levels are high, implying the existence of additional state-dependent mechanisms that counterbalance histaminergic suppression. Together, these results highlight the retina as an active target of descending neuromodulation and suggest that early vision is shaped by a dynamic interplay of multiple modulatory systems, with histamine being only one of several factors that link brain state to perception at the very first stage of vision.

## Abbreviation:

dLGN: dorsal lateral geniculate nucleus
RGCs: retinal ganglion cells
SC: superior colliculus
TMN: tuberomammillary nucleus
